# An overview of gambling in Nigeria

**DOI:** 10.1192/bji.2020.28

**Published:** 2021-05

**Authors:** Chinyere Mirian Aguocha, Sanju George

**Affiliations:** 1 Consultant Psychiatrist and Senior Lecturer (Psychiatry), Department of Internal Medicine, Imo State University, Owerri, Nigeria; 2 Professor of Psychiatry and Psychology, Rajagiri School of Behavioural Sciences and Research, Rajagiri College of Social Sciences (Autonomous), Kochi, India. Email: sanjugeorge531@gmail.com

**Keywords:** Gambling, Nigeria, policy, research, public health

## Abstract

Gambling, legal and illegal, is popular in Nigeria. Lack of stringent regulation and enforcement, coupled with the rise in online gambling opportunities, has resulted in increased gambling-related harm. There needs to be a multipronged public health strategy to address the harms of gambling and for this the government, gambling industry, policy makers and academic experts need to engage in a meaningful debate.

Nigeria is Africa's most populous country, with a population of about 185 million people. It is the seventh most populated country in the world and is also the world's twentieth largest economy. Nigeria gained independence from the British in 1960 and has since been governed by civilian leaders and military dictators. Nigeria is currently governed by a democratically elected party but is troubled by religious fundamentalist groups.

In this brief paper, we will present an overview of the gambling landscape of Nigeria.

## Gambling in Nigeria

Gambling has always existed in Nigeria but in the past it was viewed as an antisocial activity and was actively discouraged by the church, which warned against the quest for quick wealth. In the late 1990s, in Chapter 22, section 236 of the Criminal Code Act, the Nigerian government legalised certain forms of gambling in an attempt to generate tax revenues.^1^ This has made gambling more acceptable to the public, especially to the under-aged.^2^ The most popular forms of gambling in present-day Nigeria are online sports betting (e.g. football league promotions and the pools), the lottery and slot machines.^3^ Many Nigerians view gambling as a harmless leisure activity: a recent study of the Nigerian general population found that 36% of adult respondents had gambled and 53% of these people were daily gamblers.^4^ However, some argue that problem gambling in Nigeria, in the near future, will be a greater public health problem than substance misuse. Illegal gambling, especially betting on football, is extremely popular in Nigeria, although its precise scale is unknown. It is surprising that, despite the nature and scale of this problem, gambling and its related harms have not been adequately researched in Nigeria.

## Gambling laws in Nigeria

Gambling in Nigeria is regulated by the National Lottery Regulatory Commission. The National Lottery was legalised in Nigeria in 2005, under the National Lottery Act 2005.^5^ The law distinguishes between games of skill (which are legal) and games of chance (which are illegal). Legal forms of gambling include the lottery, land-based casinos and sports betting, whereas roulette, dice games and non-skilled card games are considered illegal. There is no specific provision in the law to regulate online gambling. The minimum legal age to be able to gamble in Nigeria is 18 years.

## Gambling research from Nigeria

Gambling research in Nigeria has been mainly focused on the prevalence, pattern and determinants of gambling among the different subpopulations in the country.^2,3,6^ A significant relationship has been reported with age, gender, financial strain, some personality factors and depression.^3,7^ It has been argued that monetary gain, fuelled by greed, unemployment, economic hardship and poverty are the most potent motivating factors for gambling and may act as a spring board to fuel criminality.^4^ Other less important factors are the pursuit of enjoyment, passion for sports and peer group influence.^4^ Friends who engage in gambling, gambling to gain the acceptance of friends, parental gambling and problems with authorities on account of gambling have been reported as important predictors of gambling.^2^ Studies carried out to determine attitudes towards and perception of gambling found that, although gambling was perceived as a risky activity, it was believed to yield high returns and was thus perceived as a means of earning money quickly.^8^ In spite of the laws regulating gambling in Nigeria, about 57.2% of school-age children have gambled at least once in their lifetime and 77.6% of these have gambled in the past year, with 58.3% reporting unfettered access to gambling dens.^2^

## Treatment services for problem gamblers in Nigeria

Problem gambling refers to continuous, uncontrollable gambling despite harmful negative consequences.^9^ There are no specialist treatment centres for problem or pathological gamblers in Nigeria. As awareness of gambling problems is limited among the public and among healthcare and allied professionals, most people with gambling-related issues go unrecognised and untreated. Those in whom it is identified as a problem get addiction treatment from existing substance use treatment centres and specialists. To the best of our knowledge, no Gamblers Anonymous meetings take place anywhere in Nigeria. The few very wealthy Nigerians who have gambling problems seek specialist addiction treatment, including therapy in rehabilitation clinics overseas.

## The way forward: problem gambling merits a public health prevention approach

Harms from problem gambling are multiple and often not as direct and visible as in substance addictions. A range of harms that adversely affect the individual, the family and society have been reported. These include uncontrolled gambling, sometimes fuelled by desperate attempts to win back money, financial difficulties and extreme poverty; disruption of relationships within the family, with loss of trust and failure to carry out expected responsibilities; engagement in other risky behaviours, such as excessive drinking, substance use and crime.^10^ Despite most forms of gambling being illegal in Nigeria, substantial amounts are spent on a daily basis.^4^ The rate of problem gambling is estimated to range from 1 to 8%, with 10–15% at risk of problems related to gambling.^11,12^ With the increasing influence of the West and the fast-paced technological penetration in Nigeria, it is anticipated that problems related to gambling will increase. Unfortunately, prevention approaches are currently almost non-existent.

It is against this background that it is pertinent to conceptualise an appropriate public health response to gambling in Nigeria.

We propose a three-tiered framework for such a public health response: primary, secondary and tertiary. Primary prevention focuses on preventing or postponing initiation of the first bet. Primary preventive approaches aim to make the general public aware of the risks and potential negative consequences of excessive gambling and to help them make decisions about ‘responsible’ gambling. This should be targeted mainly at preventing under-age gambling and protecting other vulnerable groups. Concerted efforts have to be made to develop age verification processes to prevent under-age online gambling. Community awareness campaigns on the dangers of gambling, together with funding and disseminating research on gambling, have to be carried out. Voluntary contributions, a statutory levy imposed on the gambling industry or the industry taking these up as part of their corporate social responsibility should be used to fund these. Regulation of advertisements and promotions of gambling by regulating the types of advert and disclosing during adverts the risks inherent in gambling, and incorporating into the civic education curriculum of primary and secondary schools the dangers of gambling, are also desirable. Government should regulate the density of gambling outfits that can be set up in a particular location and sites where they are permitted to operate. Government recognising that gambling is a public health problem, with policy development and responsibility domiciled in the Ministry of Health, would go a long way in helping this materialise. Current regulations that ban under-age gambling have to be strictly enforced by the appropriate regulatory authority. For adults who gamble, responsible gambling should be promoted. It is here that perhaps Nigeria's policy makers could look West: one example is Britain's Responsible Gambling Strategy Board, an independent panel of experts that advises the government on aspects related to responsible or safe gambling.

Secondary prevention should screen for potential problems in those already initiated into gambling. Secondary prevention measures target ‘at-risk’ and problem gamblers. It aims to ensure early diagnosis and treatment for the individual by raising awareness of healthcare professionals about gambling and by breaking down barriers to treatment-seeking. It also involves the gambling industry's self-regulation to ensure reduced harm to their clients. Clinics for treating those with gambling problems should be set up in government hospitals, and health workers in primary health clinics should be trained in the identification of problem gambling, with an appropriate referral pathway set up.

Tertiary prevention strategies include specialised psychological and other treatment interventions for gamblers and their families.

Finally, from a public health point of view, other important parameters that would help quantify the problem of gambling include the following: the rates of various psychosomatic symptoms (cardiovascular, musculoskeletal, gastrointestinal and other non-specific psychosomatic symptoms) and psychiatric problems such as depression, anxiety, substance misuse and personality disorders among problem gamblers; financial problems such as debt and bankruptcy; gambling-related crime; and interpersonal relationship problems such as neglect of the family, domestic violence and child abuse. Much more research is called for into measuring or estimating the above-noted health and social costs borne by society.

## Conclusions

There needs to be a wider debate about gambling as a public health issue in Nigeria, involving key stakeholders such as academics, healthcare professionals, policy makers and the gambling industry. Positive action is required to minimise gambling-related harm to the people of Nigeria.
